# Investigating the therapeutic efficacy of microcurrent therapy: a narrative review

**DOI:** 10.1177/20406223251361677

**Published:** 2025-08-15

**Authors:** Sarahrose Jonik, Andrew J. Rothka, Neyha Cherin

**Affiliations:** Penn State College of Medicine, Hershey, PA, USA; Penn State College of Medicine, Hershey, PA, USA; Department of Physical Medicine and Rehabilitation, The Milton S. Hershey Medical Center, Penn State College of Medicine, 500 University Drive, Hershey, PA 17033, USA

**Keywords:** chronic pain, microcurrent, rehabilitation, wound healing

## Abstract

Microcurrent (MIC) therapy is a non-invasive, low-intensive electrical modality that remains underutilized despite evidence supporting its therapeutic potential. With applications in chronic pain, wound healing, musculoskeletal injuries, deconditioning, and neuropsychological conditions, MIC offers a pain-free alternative to traditional electrotherapies. This narrative review compiles the current literature on MIC therapy, highlighting its physiological mechanisms, such as promoting cellular repair, modulating inflammation, and reducing pain, without inducing discomfort or muscle fatigue. Though more high-quality evidence is needed, this review examines the current evidence on MIC’s role in managing chronic and complex conditions across diverse healthcare environments and patient populations.

## Introduction

Microcurrent (MIC) therapy is a non-invasive treatment modality that emits subsensory electrical currents to promote healing and tissue repair. These impulses, typically delivered in the microampere (μA) range, mimic the body’s endogenous electrical signals and influence cellular processes such as adenosine triphosphate (ATP) production, protein synthesis, blood flow and oxygenation, and waste product removal. Such processes promote normal membrane function by decreasing resistance and inflammation, stimulating tissue development, and encouraging repair.^1–6^

In contrast, transcutaneous electrical nerve stimulation (TENS) units provide electrical currents in the milliampere range, which is 1000 times higher than MIC therapy, resulting in nerve depolarization, muscle contraction, fatigue, and pain. MIC therapy, by operating at significantly lower intensities, avoids these adverse effects while still offering therapeutic benefits.^7–9^

Various MIC-based and neuromuscular modalities, including MIC electrical neuromuscular stimulation (MENS), neuromuscular electrical stimulation (NMES), and functional electrical stimulation (FES), are sometimes used interchangeably in the literature. However, this review will focus, in particular, on low-intensity MIC therapy along with its underlying mechanisms, therapeutic applications, and current limitations. Given its potential in treating a wide range of conditions, including pressure injuries (PIs), burns, deconditioning, musculoskeletal and neuropathic pain, chronic pain, and mental health, the authors believe MIC is an underutilized technique that should be more widely considered in clinical practice. The purpose of this article is to evaluate the current body of evidence for the broader clinical use of MIC therapy across medical specialties and interpret the scope of MIC therapy applications, beyond direct TENS comparisons, to highlight areas where MIC may offer unique or underrecognized advantages. We conducted a comprehensive literature review using PubMed, Web of Science, the Cochrane Library, and Scopus as the primary databases. We reviewed specifically the literature that addressed clinical outcomes related to the application of MIC therapy (i.e., pain scores, healing rates, or quality-of-life metrics), and focused on one or more of the following clinical domains: rehabilitation, chronic pain, wound healing, psychiatric or neurocognitive conditions, or oncologic support.

## Mechanisms of microcurrent therapy

### Biochemical and cellular mechanisms

The therapeutic mechanisms of MIC are thought to involve a combination of electrophysiological and biochemical mechanisms. MIC may restore transmembrane potential and promote calcium influx, which activates second messenger systems, including cyclic AMP and protein kinase pathways.^6^ This stimulation has been linked to increased ATP synthesis, improved mitochondrial efficiency, as well as fibroblast and satellite cell activation to boost tissue and muscle repair.^10–15^ Furthermore, MIC has been shown to enhance endothelial nitric oxide production and angiogenesis, contributing to improved circulation and wound healing.^16^ MIC ampere intensity also serves distinct therapeutic purposes depending upon the specific current range utilized. For example, a 100–500 μA current applied to injured tissue increases satellite cells, ATP generation, amino acid transport, and protein synthesis by 30%–40% above control levels.^4^ In addition, an isolated current of 200 μA enhanced connective tissue matrix formation by stimulating fibroblast production,^12,14^ leading to granulation-tissue formation, which promotes effective initiation of the physiologic healing process in wounds such as PIs and burns.^14^

### Electrophysiological mechanism and effects

MIC therapy modulates pain by acting both peripheral and central nervous system pathways, supporting neural regeneration and functional connectivity. MIC applied via cranial electrotherapy stimulation (CES) applies alternating subsensory charge via earclip electrodes to the nervous system. CES then positively alters brain activity and modifies painful-processing regions in the brain. Data record that 42%–46% of CES currents pass to deep brain tissue via the cranial nerves.^17^ The majority of the charge registers within the thalamus, which holds a key role in the neurotransmitter homeostasis needed for pain modulation.^17^ Research utilizing functional magnetic resonance imaging and electroencephalographic (EEG) studies demonstrates that CES is able to restore neurotransmitter imbalance and activate cortical deactivation, subsequently inducing relaxed states and enhancing analgesic effects.^18–20^

While the exact mode of action is unclear, MIC is presumed to mirror Arndt–Schultz law theory. For instance, one study found that applying a 500-μA current (total applied current across the treatment site) increased ATP productivity by 500%, whereas a 5-mA current inhibited production.^6^ While most studies report total current, the current density (μA/cm^2^) is not consistently reported across the literature. This distinction is important, as the therapeutic effect likely depends on both the absolute current and electrode-skin interface area over which it is applied.^21^ This research reinforces that low-level amperage stimulates physiologic cellular activity and is beneficial for therapeutic healing, whereas high currents are detrimental.

## Clinical applications of microcurrent therapy

### Would healing and tissue repair

Current literature emphasizes the utility of MIC therapy on tissue and muscle repair, particularly for use with supplementing the PI healing process in damaged tissue.^15,22–25^ Zhao et al. discovered that MIC mimics the electrical intensity range produced by endogenous electrical tissue during physiological wound healing.^15^ Consequently, MIC application to ischemic wounds has been shown to accelerate healing at twice the rate of sham-treated wounds (standard wound care).^26^ Currents greater than 300 μA have demonstrated increased healing rates for stage 2 and 3 chronic decubitus PI, while intensities between 50 and 100 μA reduced wound closure time in thermal injuries by 36%.^27^ Furthermore, extensive research demonstrates that MIC minimizes wound area in PI and burns, decreases healing time, and reduces pain by mitigating inflammation and stimulating angiogenesis.^15,16,22,24,25,28^ A recent meta-analysis confirmed MIC to be more effective than standard wound care, while also providing analgesic relief for patients with cutaneous wounds.^22^ Therefore, the implementation of MIC therapy for high-risk patients, such as those with spinal cord injury (SCI), has potential to amplify the healing process and prevent the progression of chronic wounds.

The various forms of MIC application, including NMES, MENS, and FES, have demonstrated efficacy in improving muscle fiber recovery, reducing delayed-onset muscle soreness, and enhancing post-exercise recovery for both injured and healthy tissue.^6,29^ For instance, MENS has been shown to improve circulation, reduce inflammation, and expedite tissue repolarization, ultimately minimizing pain and shortening recovery time.^30–32^ Additionally, the ability for MIC therapy to strengthen cellular communication and enzymatic functioning positively influences deconditioning, weakness, and aging, making it particularly relevant for elderly or immobilized patients.^33^ For example, elderly patients receiving MIC therapy demonstrated amplified physical performance, and studies have found diminished muscle atrophy in immobilized tissue at low-intensity currents.^2,33–35^

### Musculoskeletal conditions

The therapeutic effects of MIC extend to both acute and chronic musculoskeletal dysfunction as well. A double-blinded randomized controlled trial found that MIC therapy increased functionality and decreased pain in acute knee injuries.^36^ Moreover, post-knee arthroplasty patients treated with MIC-therapy-induced gelatinous synthesis, demonstrating improved ROM and enhanced tissue recovery.^27^ Similar benefits have been observed in chronic conditions, where MIC therapy repolarizes skeletal muscle tissue, loosens adhesions, and stimulates growth factor activity.^1^ Studies have also shown that MIC therapy improves pain and functionality in knee osteoarthritis, temporomandibular joint disorders, and restricted ROM secondary to scar tissue or congenital conditions.^37–39^ A systematic review comparing 3 h of daily active MIC therapy to a sham placebo for knee pain found a significant increase in pain reduction in the MIC group, particularly by the third week of the study (*p* < 0.01).^36,40^ Importantly, MIC therapy has also been safely implemented in pediatric populations, improving functionality in conditions such as congenital torticollis and femoral anteversion.^41–44^

### Systemic and oncologic applications

There is notable promise of MIC therapy in addressing systemic conditions such as deconditioning and cancer-related impairments.^45^ A Cochrane review found NMES to be safe and effective for slowing muscle atrophy chronic disease states and muscle disuse in adults.^35^ Other studies indicate that MIC therapy enhances blood flow, reduces radiation-induced fibrosis, and alleviates lymphedema, supporting oncologic patients in ameliorating cervical ROM restrictions and preventing further complications.^45,46^ These findings underscore the modality’s utility in mitigating morbidity factors that impede patient progress in physical and occupational therapies, ultimately expediting functional gains and return to daily activities.

Another significant advantage of MIC therapy lies in its application for pain relief, particularly in patients with chronic conditions such as fibromyalgia, chronic regional pain syndrome, and Ehlers–Danlos syndrome. Tissue trauma and inflammation often impedes electrical conductance, resulting in nerve dysfunction, contributing to a clinical picture of widespread pain. Studies have demonstrated improvements in pain scores for patients with fibromyalgia and reduced pain intensity in SCI populations, without the systemic side effects associated with pharmacological treatments.^47–49^

### Neurological and psychiatric applications

Finally, MIC therapy’s neuromodulatory effects extend to neurological and psychiatric disorders through CES, as well. A network meta-analysis and double-blind placebo control trial on fibromyalgia patients reported MIC as highly effective at improving pain scores.^8^ Similar promising results among the SCI population have been reported as well. For example, Capel discovered MIC minimized pain intensity in the chronic SCI population as well.^50^ Another study focusing on SCI neuropathic pain demonstrated CES to be statistically significant in improving pain scores.^47,51^

CES has also been shown to enhance neurotransmitter flow, improve neurocognitive activity, and regulate brainwave patterns on EEG, offering therapeutic potential for conditions like anxiety, depression, and insomnia.^17,52–54^ Studies hypothesize that CES diminishes negative responses to worry and conversely boosts positive attention.^55,56^ While some data on CES’s efficacy remain inconsistent with roughly half of data demonstrating improvement or reduced sleep latency, high-quality studies have demonstrated benefits for reducing anxiety symptomatology and improving depression symptoms.^53,57^

CES is FDA approved for treating anxiety, depression, and insomnia, highlighting its low-risk profile and accessibility.^55^ Furthermore, less than 1% of patients report experiencing adverse effects of headache, skin irritation, and dizziness.^55^ Ultimately, CES can provide a valuable adjunctive therapy in clinical settings for improving quality of life, particularly in patients experiencing significant mental health challenges due to their medical conditions.

## Limitations of microcurrent therapy

Despite growing interest in MIC therapy, there are limitations that hinder its wider spread clinical adoption. Practical barriers exist, including cost and availability of equipment, lack of insurance reimbursement, and minimal awareness of its utility compared to alternative electrotherapy. At present, another major limitation is the inconsistency in clinical evidence, as many studies have small sample sizes, limited controls, and variable patient populations and treatment parameters. Such variability makes establishing standardized dosing guidelines or comparing results across studies.

Future investigations should not only assess efficacy, but also explore economic viability, particularly in populations with high care burden or limited response to conventional treatments. As healthcare continues to prioritize value-based care, the long-term benefits of MIC may ultimately prove both clinically and economically justified in targeted patient populations.

## Conclusion

Despite a growing body of evidence highlighting its therapeutic potential, MIC therapy remains underutilized across many areas of medicine. Its subsensory, low-intensity electrical currents offers a non-invasive, pain-free alternative to traditional electrotherapies like TENS, without triggering muscle contraction or discomfort. MIC therapy shows promise as an adjunctive modality capable of supporting tissue repair, reducing inflammation, and modulating pain, particularly in complex, chronic, or refractory conditions. From supporting tissue regeneration in wound care and improving musculoskeletal recovery, to enhancing outcomes in neurological and psychiatric care, MIC therapy presents a low-risk intervention with broad clinical relevance. Although barriers such as cost and accessibility may limit widespread adoption, MIC’s therapeutic potential to enhance functional outcomes and reduce symptoms burden in vulnerable patient populations makes it a compelling candidate for further investigation. Future research should focus not only on establishing efficacy through high-quality trials, but also on evaluating cost-effectiveness, optimizing treatment protocols, and addressing barriers to adoption. As healthcare continues to emphasize patient-centered, value-based care, MIC therapy has the potential to expand the therapeutic landscape within rehabilitation and pain management strategies across diverse patient populations.

## Declarations

**Ethics approval and consent to participate:** Not applicable.

**Consent for publication:** Not applicable.

**Availability of data and materials:** Not applicable.
